# Effects of Resveratrol Supplementation and Exercise Training on Exercise Performance in Middle-Aged Mice

**DOI:** 10.3390/molecules21050661

**Published:** 2016-05-18

**Authors:** Nai-Wen Kan, Chin-Shan Ho, Yen-Shuo Chiu, Wen-Ching Huang, Pei-Yu Chen, Yu-Tang Tung, Chi-Chang Huang

**Affiliations:** 1 Center for Liberal Arts, Taipei Medical University, Taipei 11031, Taiwan; kevinkan@tmu.edu.tw; 2 Graduate Institute of Sports Science, National Taiwan Sport University, 250 Wenhua 1st Rd., Guishan Township, Taoyuan County 33301, Taiwan; kilmur23@ntsu.edu.tw (C.-S.H.); 1021301@ntsu.edu.tw (Y.-S.C.); magicpica521@gmail.com (W.-C.H); 1010206@ntsu.edu.tw (P.-Y.C.); 3 Department of Orthopedic Surgery, Shuang Ho Hospital, Taipei Medical University, New Taipei City 23561, Taiwan; 4 School of Nutrition and Health Sciences, Taipei Medical University, Taipei City 11031, Taiwan

**Keywords:** resveratrol, anti-fatigue, exercise performance

## Abstract

Resveratrol (RES) has antioxidative, anti-inflammatory, anticancer, antidiabetic, antiasthmatic, antalgic, and anti-fatigue activities. Exercise training (ET) improves frailty resulting from aging. This study evaluated the effects of a combination of RES supplementation and ET on the exercise performance of aged mice. C57BL/6J mice (16 months old) were randomly divided into four groups: an older control group (OC group), supplementation with RES group (RES group), ET group (ET group), and a combination of ET and RES supplementation group (ET+RES group). Other 10-week-old mice were used as a young control group (Y-Ctrl group). In this study, exercise performance was evaluated using forelimb grip strength and exhaustive swimming time, as well as levels of plasma lactate, ammonia, glucose, and creatine kinase after an acute swimming exercise. Our results showed that the forelimb grip strength of mice in the ET+RES group was significantly higher than those in the OC, RES, and ET groups (by 1.3-, 1.2-, and 1.1-fold, respectively, *p* < 0.05), and exhibited no difference with the Y-Ctrl group. The endurance swimming test showed that swimming times of the ET and ET+RES groups were significantly longer than those of the OC and RES groups. Moreover, plasma lactate and ammonia levels of the ET + RES group after acute swimming exercise were significantly lower compared to the OC group (*p* < 0.05). Thus, it was suggested that by combining RES supplementation with ET for 4 weeks, the muscle strength and endurance performance of aged mice were significantly improved compared to the single intervention with either RES or ET alone. This combination might help shorten the extent of deterioration accompanying the aging process.

## 1. Introduction

Fatigue is usually classified into mental and physical fatigue. In a fatigued status, subjects find it difficult to initiate or sustain voluntary activities. Physical fatigue is thought to be accompanied by a deterioration in body functions [1]. There are some theorized mechanisms of exercise-induced fatigue, including exhaustion theory, clogging theory, mutation theory, homeostasis disturbance theory, and protective inhibition radical theory [2]. In addition, exhaustive or intensive exercise can lead to the accumulation of excess reactive free radicals that result in tissue damage [3].

Regular exercise training (ET) helps improve human physiological performance, and prevent metabolic syndrome, heart disease, cardiovascular disease (CVD), hypertension, type 2 diabetes, obesity, and other diseases associated with aging [4,5,6,7]. On the other hand, the intake of natural antioxidants is associated with low incidences of cancer, CVD, diabetes, and other diseases associated with aging [8]. Thus, scientists have begun to explore natural antioxidant products to reduce exercise-induced oxidative damage and fight against fatigue [9]. Recently, many studies showed that resveratrol (RES) possesses various pharmacological effects, such as anti-inflammatory properties, antioxidant properties, cardiovascular protection, chemoprevention of cancer, and anti-fatigue [10,11,12]. However, to the best of our knowledge, there is no prior report on the effects of a combination of RES and ET against illness and the aging-induced decrease in exercise performance. This study was designed to evaluate the effects of a combination of RES supplementation and ET on the exercise performance of aged mice.

## 2. Results

### 2.1. Effect of 4-Week RES Supplementation and ET on Body Weight, Food Intake, Water Intake, and Tissue Changes

Results of body weight, food intake, and water intake are shown in Table 1. One-way ANOVA results indicated that there were no significant differences in the body weight, water intake, or tissue weights including the liver, lungs, kidneys, muscles, and BAT of mice among the young control group (Y-Ctrl group), older control group (OC group), supplementation with RES group (RES group), ET group (ET group), and a combination of ET and RES supplementation group (ET+RES group). The results obtained herein were similar to those obtained by Wu *et al.* [12], who found that administration of RES (25 mg/kg) produced no statistical difference in body weight or tissue weights compared to the vehicle control group. Furthermore, our results differed from those obtained by Chen *et al.* [13], who found that the relative liver and BAT weights (%) were higher in the ET group than the OC group. This discrepancy may have been due to mice at different ages and of different species being tested. Accordingly, the food intake of the RES, ET, and ET+RES groups decreased by 17%, 6%, and 13%, respectively, compared to the OC group (*p* < 0.05).

### 2.2. Effect of 4-Week RES Supplementation and ET on Forelimb Grip Strength

**Figure 1:**
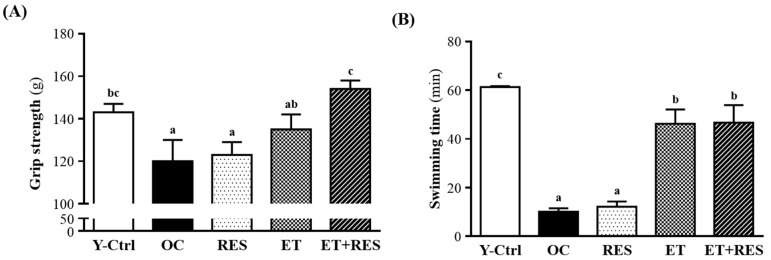
Effect of a 4-week resveratrol (RES) supplementation and exercise training (ET) on (**A**) forelimb grip strength and (**B**) endurance swimming performance in mice. Mice were pretreated with the vehicle, RES, ET, and ET+RES for 28 days. Data are the mean ± SEM. Different letters indicate a significant difference at *p* < 0.05 according to a one-way ANOVA. Y-Ctrl, a young control group; OC, an older control; RES, a supplementation with RES group; ET, a ET group; ET+RES, a combination of ET and RES supplementation group.

The grip strength was higher in the RES, ET, and ET+RES groups than the OC group (123 ± 6, 135 ± 7, and 154 ± 4 *vs.* 120 ± 10 g) (*p* < 0.05) (Figure 1A). On the other hand, compared to the OC group the relative absolute grip strength in the ET, and ET+RES groups significantly increased by 13%, and 28%, respectively. Therefore, ET combined with RES supplementation significantly increased the exercise performance. However, there were no significant differences in the forelimb grip strength between RES and OC groups. At the higher RES doses, results also showed that exercise performance did not significantly increase but the cognitive performance was significantly elevated with RES supplementation [14]. In our study, mice were pretreated with 25 mg RES/kg for 28 continuous days, and therefore this may have been more than the optimal range for endurance capacity.

### 2.3. Effect of 4-Week RES Supplementation and ET on an Exhaustive Swimming Test

Swimming to exhaustion is an animal model used to directly measure anti-fatigue effects. The model gives a high reproducibility to evaluate the endurance capacity of mice [15]. A longer swimming time is interpreted as a reduced susceptibility to fatigue. There was no significant difference in swimming times among the OC and RES groups. The results obtained herein were similar to those obtained by Wu *et al.* [12], who found that swimming times of mice pretreated with the vehicle, or 25, 50, or 125 mg RES/kg for 21 continuous days were significantly longer by 2.28-fold with 25 mg RES/kg compared to the vehicle treatment. However, there were no significant differences in swimming times among the vehicle, 50 mg RES/kg, and 125 mg RES/kg groups. These data indicate that selective concentrations of RES may differently contribute to physiological activities.

In addition, mice in both the ET and ET+RES groups exhibited prolonged swimming times. As shown in Figure 1B, the exhaustion time of the ET group was 46 min (359% greater than that of the OC group); the exhaustion time of the ET+RES group was 47 min (364% greater than that of the OC group) (*p* < 0.05), indicating that ET and ET+RES groups exhibited an anti-fatigue effect. The results obtained herein were similar to those obtained by Chen *et al.* [13], who found that ET significantly increased the absolute and relative grip strengths, by 1.19- (*p* = 0.0023) and 1.26-fold (*p* < 0.0001), respectively, compared to that of the sedentary control group. Thus, at different ages or different species, ET increased both physical performances of forelimb grip strength and exhaustive swimming exercise. To explore the mechanism, some biochemical parameters including lactase, ammonia, CK, and glucose were determined in mice after they had swum for 15 min.

### 2.4. Effect of 4-Week RES Supplementation and ET on Lactate, Ammonia, CK, and Glucose after a 15-min Swimming Test

**Figure 2:**
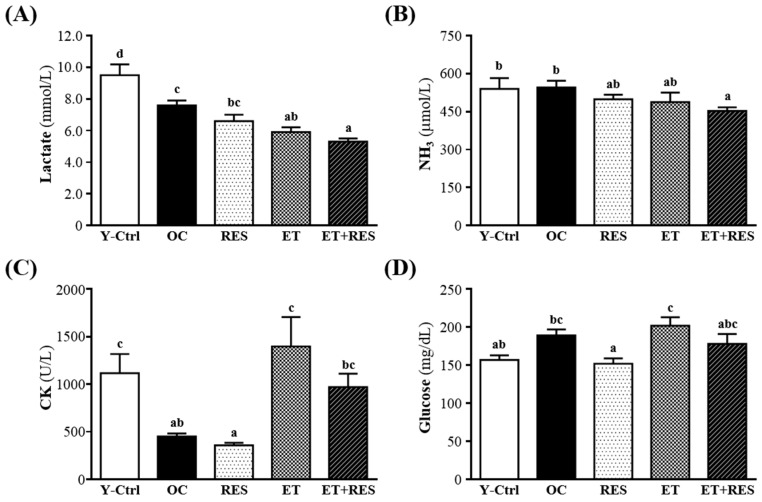
Effect of 4-week resveratrol (RES) supplementation and exercise training (ET) on serum levels of (**A**) lactate; (**B**) ammonia (NH_3_); (**C**) creatine kinase (CK); and (**D**) glucose after a 15-min swim test. Mice were pretreated with the vehicle, RES, ET, and ET+RES for 28 days. Data are the mean ± SEM. Different letters indicate a significant difference at *p* < 0.05 according to a one-way ANOVA. Y-Ctrl, a young control group; OC, an older control; RES, a supplementation with RES group; ET, a ET group; ET+RES, a combination of ET and RES supplementation group.

Blood lactate is the glycolysis product of carbohydrates under anaerobic glycolysis, and glycolysis is the main energy source for short-term high-intensity exercise [16]. The increased lactate level further reduces pH values of muscle tissues and blood, and the phenomenon can induce various side effects of several biochemical and physiological processes [9]. Therefore, blood lactate is an important blood biochemical parameter related to fatigue [17]. After swimming, levels of blood lactate of the RES, ET, and ET+RES groups were significantly lower by 12%, 22%, and 30%, respectively, than that of the OC group (*p* < 0.05) (Figure 2A). Thus, regardless of whether ET, RES, or the combination of RES and ET were applied, they all reduced fatigue.

Ammonia, the metabolite of proteins and amino acids, is linked to fatigue [18]. The increase in ammonia in response to exercise can be managed by the use of amino acids or carbohydrates that interfere with ammonia metabolism [19]. The increase in the ammonia level is related to both peripheral and central fatigue during exercise [9]. Therefore, the blood ammonia level is an important blood biochemical parameter related to fatigue. After RES supplementation, ET, or the combination of RES supplementation and ET in mice for 28 days, serum ammonia levels were lower in the RES (499 ± 18 mg/dL; 8% lower than that of the OC group), ET (488 ± 37 mg/dL; 11% lower than that of the OC group), and ET+RES groups (453 ± 14 mg/dL; 17% lower than that of the OC group) than the OC group (546 ± 26 mg/dL) after the swimming test. Thus, it is suggested that when combining RES supplementation and ET for 4 weeks, the ammonia level of aged mice after the swimming test might be significantly improved compared to the single intervention of either RES or ET alone.

A high-intensity exercise challenge can physically or chemically cause tissue damage. It can cause sarcomeric damage and muscular cell necrosis [20]. Muscle cells release CK into the blood which indicates that muscle damage has occurred or is occurring. Clinically, CK is known to be an accurate indicator of muscle damage. As shown in Figure 2C, the serum CK level of the RES group (357 ± 27 U/L) was lower compared to the OC control (450 ± 33 U/L). However, those of the ET (1398 ± 307 U/L) and ET+RES groups (971 ± 140 U/L) were significantly higher compared to the OC control. CK is from the heart but they cannot rule out the possibility that some or much of the CK could have come from exercising skeletal muscles. In this study, we supposed ET may damage skeletal muscle cells resulting in increased total serum CK. However, RES treatment can reduce the serum CK level, indicating that RES treatment may ameliorate ET-induced sarcomeric damage or muscular cell necrosis.

Regulation of blood glucose homeostasis plays an important role in prolonging endurance exercise [21]. Hypoglycemia can inhibit the active functioning of the brain during exercise, and this often leads to the inability to continue exercising [22]. Thus, blood glucose homeostasis is an important blood biochemical parameter related to fatigue. As shown in Figure 2D, the blood glucose level of the ET group was actually slightly higher than that of the OC group. This indicates that exercise can regulate blood-glucose levels. However, the blood glucose level of the RES group was significantly 20% lower than that of the vehicle control group (*p* < 0.05). Thus, we supposed that RES may promote glucose utilization by peripheral tissues and has a glucose-lowering action.

**Figure 3:**
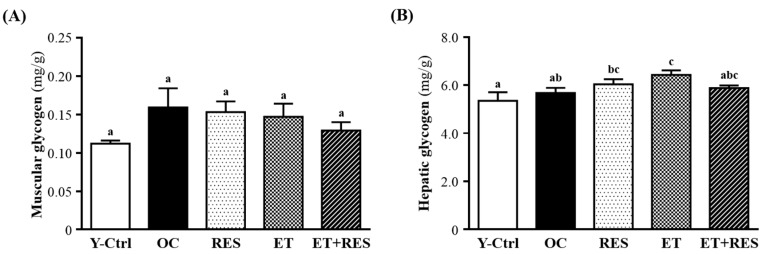
Effect of 4-week resveratrol (RES) supplementation and exercise training (ET) on levels of (**A**) muscular and (**B**) hepatic glycogen. Mice were pretreated with the vehicle, RES, ET, and ET+RES for 28 days. Data are the mean ± SEM. Different letters indicate a significant difference at *p* < 0.05 according to a one-way ANOVA. Y-Ctrl, a young control group; OC, an older control; RES, a supplementation with RES group; ET, a ET group; ET+RES, a combination of ET and RES supplementation group.

Muscle glycogen concentration can be greatly changed by training status, exercise routines and diet. As shown in Figure 3A, there is no statistically significantly different among all groups. It revealed that the resynthesis of muscle glycogen after exercise-induced depletion is equal. Liver glycogen is also an index of fatigue. The role of hepatic glycogen is to complement the consumption of blood glucose to maintain blood glucose in the physiologic range. Fatigue occurs when liver glycogen is mostly consumed [23]. As shown in Figure 3B, liver glycogen levels of the RES (6.04 ± 0.21 mg/g; 6% higher than that of the OC group), ET (6.43 ± 0.19 mg/g; 13% significantly higher than that of the OC group), and ET+RES groups (5.88 ± 0.11 mg/g; 3% higher than that of the OC group) were higher than that of the OC group (5.68 ± 0.21 mg/dL). RES, fasting, and calorie restriction can activate the expression and biological activity of SIRT1 which plays a key role in regulating mitochondrial biogenesis and energy metabolism in different related tissues such as the liver, skeletal muscles, adipose tissues, and the pancreas [24].

PGC-1α activation by SIRT1 in liver cells can result in downregulation of glycolytic pathways and upregulation of gluconeogenic pathways [25]. In addition, resveratrol improves mitochondrial function and protects against metabolic disease by activating SIRT1 and PGC-1α [26]. Therefore, we found that glycogen contents of liver tissues did not show significant differences between the OC and RES groups. In addition, the results also showed that the liver glycogen level of the ET group was significantly higher than that of the OC group, indicating that ET may improve the metabolic control of exercise and activation of energy metabolism. The data agreed with previous findings [22].

### 2.5. Effect of 4-Week RES Supplementation and ET on Biochemical Assessments

**Figure 4:**
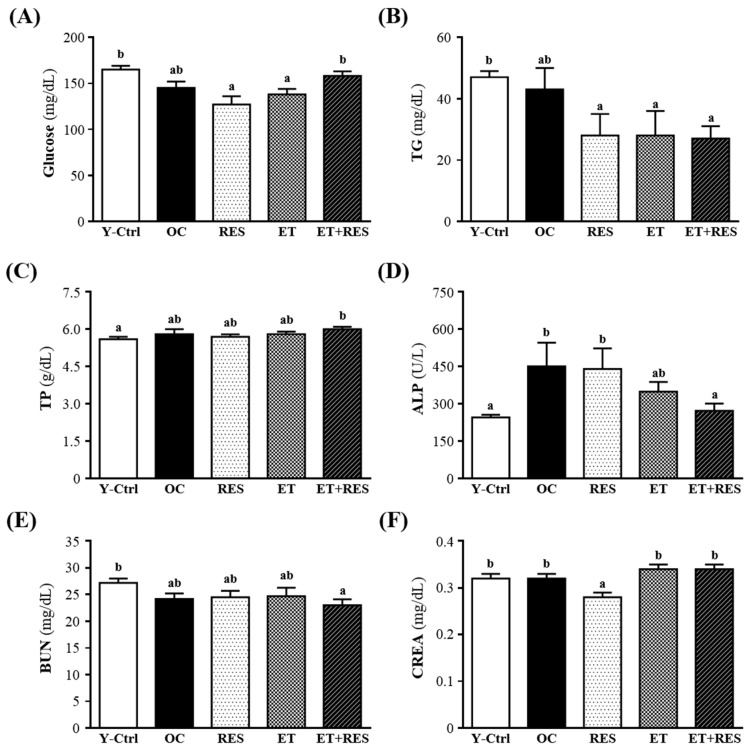
Effect of 4-week resveratrol (RES) supplementation and exercise training (ET) on biochemical assessments at rest, including (**A**) glucose; (**B**) triglyceride (TG); (**C**) total protein (TP); (**D**) alkaline phosphatase (ALP); (**E**) blood urea nitrogen (BUN); and (**F**) creatinine (CREA) levels in serum. Mice were pretreated with the vehicle, RES, ET, and ET+RES for 28 days. Data are the mean ± SEM. Different letters indicate a significant difference at *p* < 0.05 according to a one-way ANOVA. Y-Ctrl, a young control group; OC, an older control; RES, a supplementation with RES group; ET, a ET group; ET+RES, a combination of ET and RES supplementation group.

In the present study, we observed beneficial effects of RES supplementation and ET on the exhaustive exercise challenge and measured other physiological effects after 28 days of RES supplementation and ET. Biochemical data from sera of the Y-Ctrl, OC, RES, ET, and ET+RES groups are shown in Figure 4. TG contents of the RES (28 ± 7 mg/dL), ET (28 ± 8 mg/dL), and ET+RES groups (27 ± 4 mg/dL) were significantly lower compared to that of the OC group (43 ± 7 mg/dL). The ET+RES group exhibited a lower ALP (272 ± 29 U/L) level compared to the OC group (451 ± 95 U/L). The creatine level of the RES group (0.28 ± 0.01 mg/dL) was 12.5% higher than that of the OC group (0.32 ± 0.01 mg/dL). And there were no significant differences in glucose, TP, or BUN of mice in the RES, ET, and ET+RES groups compared to the OC group.

However, Chen *et al.* [13], found that the serum level of ALP was higher in the ET group than in the OC group, by 1.19-fold (*p* = 0.0089), whereas levels of TP, creatinine, and TG were lower in the ET group than those in the OC group, by 8.7% (*p* < 0.0001), 11.4% (*p* = 0.0039), and 60.6% (*p* < 0.0001), respectively. This discrepancy may have been due to the mice at different ages or different species being tested.

### 2.6. Effect of 4-Week RES Supplementation and ET on Histopathological Evaluation of Tissues

**Figure 5:**
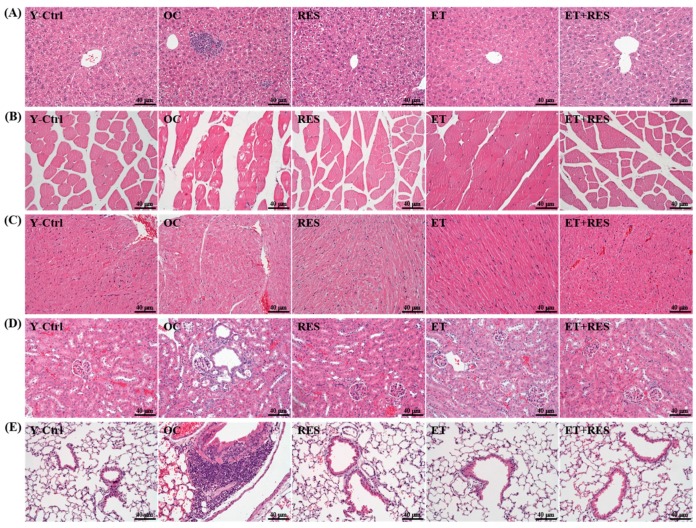
Effect of 4-week resveratrol (RES) supplementation and exercise training (ET) on histopathological evaluation of tissues, including (**A**) liver; (**B**) muscle; (**C**) heart; (**D**) kidney, and (**E**) lung. Mice were pretreated with the vehicle, RES, ET, and ET+RES for 28 days. Specimens were photographed with a light microscope (Olympus BX51). (Magnification: ×200, Scale bar, 40 μm). Y-Ctrl, a young control group; OC, an older control; RES, a supplementation with RES group; ET, a ET group; ET+RES, a combination of ET and RES supplementation group.

Figure 5 shows that multifocal necrosis, inflammatory cell infiltration and bile accumulation of liver tissue; muscle fiber abnormal of muscle tissue; renal tube regeneration basophilic of kidney tissue; and PAM proliferation of lung tissue were observed in the OC group. In addition, the other four groups (Y-Ctrl, RES, ET, and ET+RES groups) did not differ according to histological observations of the liver, muscle, heart, kidney, and lung. Thus, regardless of whether ET, RES, or the combination of RES and ET were applied, they all reduced histopathological changes.

## 3. Discussion

RES (*trans*-3,4′,5-trihydroxystilbene), which belongs to the stilbene class of polyphenolic compounds, is mainly found in skins of red grapes and has various pharmacological activities including antioxidative, anti-inflammatory, anticancer, antidiabetic, anti-asthmatic, and antalgic activities [27,28,29,30,31,32]. In addition, relatively few studies have directly addressed the possible anti-fatigue functions of RES [12]. Moderate exercise is beneficial to the heart and lung functions, improving exercise performance, and as preventive medicine helpful in reducing the incidence of chronic disease [33]. High-intensity workouts, ET, and athletic competition affect the body’s hemostasis, which causes pathological syndromes. Physiological functions such as oxidative systems and important tissues are affected by long-term and high-intensity exercise that exceeds the body’s endurance [34,35]. In this study, we investigated the beneficial synergistic effects of RES supplementation and swimming ET on exercise performance, biochemical profiles, and pathological responses after long-term supplementation.

Forelimb grip strength is a routine physical examination test. Our previous study found that muscle strength was positively correlated with forelimb grip strength [12]. In this study, we found greater grip strength in the RES, ET, and ET+RES groups than in the OC group. These data agreed with previous results showing that RES can improve muscle strength. In addition, there are beneficial synergistic effects of RES and long-term aerobic ET on muscle strength. To examine the effectiveness of ET and/or RES supplementation on improving the exercise endurance capacity, all animals underwent a swim-to-exhaustion exercise test. Compared to RES supplementation alone, ET and RES supplementation significantly prolonged the swimming time to exhaustion, so ET alone significantly improved the exercise endurance of test animals after 8 weeks of ET. Simultaneously, RES supplementation did not prolong the swimming time, but it did increase antioxidant activity and protect against long-term ET-induced acute-phase responses. A previous report demonstrated that long-term ET can imbalance the antioxidant status and result in acute tissue injury or muscle fatigue [36]. In addition, oxidative stress can induce muscle damage and affect protein metabolism in muscles [37]. RES supplementation can thus inhibit the oxidation of muscle proteins induced by ET. RES supplementation after ET may reduce the resulting long-term ET physiologic fatigue, thereby contributing to improved exercise performance.

In addition, plasma lactate and ammonia levels of ET+RES groups decreased after acute swimming exercise compared to those of the OC group. Compared to RES supplementation, ET and RES supplementation significantly decreased plasma lactate. However, it was interesting that the CK level of the ET and ET+RES groups increased compared to the OC control. During intensive exercise or long-term training, biochemical variables are significantly altered. Acute aerobic physical exercise, such as exhaustive swimming exercise, might significantly elevate the activity of traditional biomarkers such as aspartate aminotransferase (AST), CK, and bilirubin [12]. These post-exercise biomarkers of cardiac and skeletal muscle damage remain elevated at 24 h after a workout [38]. Many kinds of nutrient supplements, within different experimental models, were found to have effective protective effects on these biomarkers. In this study, we found that with long-term ET combined with RES, the CK level was lowered, indicating that RES has possible protective effects.

## 4. Materials and Methods

### 4.1. Materials

A commercially available supplement, *trans*-RES, was provided by Biotivia Bioceuticals LLC (Fenix Biotechnology, New Taipei City, Taiwan).

### 4.2. Animals and Treatment

Male C57BL/6J mice were purchased from BioLASCO (A Charles River Licensee Corp., Yilan, Taiwan). All animals were fed a chow diet (no. 5001; PMI Nutrition International, Brentwood, MO, USA) and distilled water *ad libitum*, and were maintained on a regular cycle (12-h light/dark) at room temperature (24 ± 2 °C) and 60%~70% humidity. The bedding was changed and cleaned twice per week. All animal experimental protocols were approved by the Institutional Animal Care and Use Committee (IACUC) of National Taiwan Sport University, and the study conformed to the guidelines of the protocol IACUC-10205 approved by the IACUC ethics committee.

C57BL/6J mice at 16 months old were randomly divided into four groups: (1) an older control without ET group (OC group); (2) supplementation with RES without ET group (RES group); (3) ET group (ET group); and (4) combination of ET and RES supplementation group (ET+RES group). In addition, other 10-week-old mice were used as a young control without ET group (Y-Ctrl group). 2.5 mg/kg BW of human could increase longevity through induction of proteins including Sirt, FoxO and PBEF, as well as alleviate cardiac dysfunction in streptozotocin-induced diabetes by up regulating nitric oxide, thioredoxin, HO-1, MnSOD activity [39]. Thus, in this study we treat the mice with 25 mg/kg BW, equivalent to a human dose of 2.7745 mg/kg BW. Oral gavage was used to administer 25 mg/kg mice/day RES in the RES and ET+RES groups once a day for 28 consecutive days. The OC, ET, and Y-Ctrl groups received the same volume of distilled water equivalent to an individual’s body weight. After each treatment, all groups of mice were allowed to rest for 30 min. The ET and ET+RES groups were forced to swim for 30 min to become accustomed to swimming. The OC and RES groups were confined in a plastic tank with no water. Food intake and water consumption were monitored daily, and the body weight was recorded weekly.

### 4.3. Forelimb Grip Strength

A low-force testing system (Model-RX-5, Aikoh Engineering, Nagoya, Japan) was used to measure the forelimb absolute grip strength as we previously described [9], and the maximal force (grams) was recorded. A force transducer equipped with a metal bar (2 mm in diameter and 7.5 cm long) was used to measure the amount of tensile force by each mouse. We grasped the mouse at the base of the tail and lowered it vertically toward the bar. The mouse was pulled slightly backwards by the tail while the two paws (forelimbs) grasped the bar, which triggered a “counter pull.” This grip strength meter recorded the grasping force in grams. Grip strength was measured 1 h after the last treatment was administered. The maximal force (grams) exerted by the mouse counter pull was used as the forelimb grip strength.

### 4.4. Exhaustive Swimming Test

Mice were individually placed in a columnar swimming pool (65 cm high with a radius of 20 cm) with 40 cm of water depth maintained at 24 ± 1 °C. A weight equivalent to 5% of the body weight was attached to the root of the tail, and the swimming time was recorded from the beginning to exhaustion for each mouse in the various groups. Exhaustion was determined by observing failure to swim, and the swimming period was regarded as the time spent by the mouse floating in the water, struggling, and making necessary movements until strength exhaustion and drowning. When a mouse was unable to remain on the water surface, it was assessed. The swimming time from beginning to exhaustion was used to evaluate the endurance performance.

### 4.5. Fatigue-Associated Biochemical Indices

After 4 weeks of the intervention, mice underwent a 15-min swimming test without weight loading to evaluate fatigue-associated biochemical variables as in our previous studies [9,12]. Blood samples were immediately collected after the swimming exercise. Serum was collected by centrifugation at 1500× *g* and 4 °C for 10 min. Lactate, ammonia, creatine kinase (CK), and glucose levels were determined by use of an autoanalyzer (Hitachi 7060, Hitachi Ltd., Tokyo, Japan).

### 4.6. Blood Biochemical Assessments

At the end of the experiments, all mice were killed by 95% CO_2_ asphyxiation and blood was withdrawn by cardiac puncture after an 8-h fast. Serum was collected by centrifugation, and levels of glucose, triacylglycerol (TG), total protein (TP), alkaline phosphatase (ALP), blood urine nitrogen (BUN), and creatinine (CREA) were assessed using an autoanalyzer (Hitachi 7060).

### 4.7. Tissue Glycogen Determination

The muscles and liver were excised and weighed for a subsequent glycogen content analysis. The method of glycogen analysis was described in our previous studies [9].

### 4.8. Histological Staining of Tissues

Different tissues were collected and fixed in 10% formalin after mice was sacrificed. After the formalin fixed, tissues were then embedded in paraffin and cut into 4-μm thick slices for morphological and pathological evaluations. Tissue sections were stained with hematoxylin and eosin (H & E) and examined by light microscopy with a CCD camera (BX-51, Olympus, Tokyo, Japan) by a clinical pathologist.

### 4.9. Statistical Analyses

All results are expressed as mean ± SEM (*n* = 8). The significance of difference was calculated by a one-way ANOVA with Duncan’s test, and values of *p* < 0.05 were considered significant.

## 5. Conclusions

In this study, we provide evidence that when combining RES supplementation with ET for 4 weeks, the muscle strength and endurance performance of aged mice significantly improved compared to a single intervention with either RES or ET alone. According to physical, biochemical and pathological examinations, from non-invasive to invasive tests, our findings provide evidence that supports the effects of a combination of RES supplementation and ET on the exercise performance. We admit that we do not have sufficient budget and advanced technology in our education- and application-oriented but not research center. Indeed, we have collected and preserved several important tissues such as heart and muscle tissue and look forward to cooperation with researchers on investigating molecular mechanisms or networks using a proteomic or metabolomic approach. For future investigations, RES ET+RES could be used in humans for protective and health purposes. We also provide the basic safety evidence from pathological observations and assessments. This study suggests alternative uses of RES as a nutrient supplement worthy of good health considerations.

## Figures and Tables

**Table 1:** Effects of 4-week resveratrol (RES) supplementation and exercise training (ET) on the body weight (BW), diet intake, water intake, and tissue changes in mice.

Parameter	Y-Ctrl	OC	RES	ET	ET+RES
Final BW (g)	30.30.3	31.70.3	32.10.5	31.10.9	32.30.4
Diet intake (g/mouse/day)	4.50.1 ^d^	4.20.1 ^d^	3.50.0 ^a^	4.00.1 ^c^	3.70.0 ^b^
Water (g/mouse/day)	6.10.2 ^a^	6.80.1 ^ab^	7.00.3 ^b^	6.90.2 ^b^	6.10.2 ^a^
Liver (g)	1.450.04 ^a^	1.420.05 ^a^	1.490.08 ^a^	1.300.09 ^a^	1.350.02 ^a^
Kidney (g)	0.390.02 ^a^	0.410.02 ^a^	0.440.02 ^a^	0.410.04 ^a^	0.440.01 ^a^
Heart (g)	0.240.02 ^b^	0.230.01 ^ab^	0.260.02 ^b^	0.180.02 ^a^	0.220.02 ^ab^
Lung (g)	0.350.04 ^a^	0.310.04 ^a^	0.350.03 ^a^	0.320.06 ^a^	0.330.04 ^a^
Muscle (g)	0.330.01 ^a^	0.290.01 ^a^	0.290.01 ^a^	0.290.02 ^a^	0.310.01 ^a^
BAT (g)	0.080.00 ^a^	0.070.01 ^a^	0.080.01 ^a^	0.100.02 ^a^	0.110.01 ^a^

Data are the mean ± SEM. Different letters in a given row indicate a significant difference at *p* < 0.05 according to a one-way ANOVA. Y-Ctrl, a young control group; OC, an older control; RES, a supplementation with RES group; ET, a ET group; ET+RES, a combination of ET and RES supplementation group; BAT, brown adipose tissue.
